# Continuous vs. intermittent vancomycin infusion in critically ill patients: a systematic review and meta-analysis

**DOI:** 10.3389/fmed.2026.1951699

**Published:** 2026-10-13

**Authors:** Yuanyuan Zhong, Peng Hua, Ling Zhang, Ruixiang Chen, Yuzhen Ding, Yan Sun

**Affiliations:** 1 Department of Pharmacy, The Third People’s Hospital of Yunnan Province, The Second Affiliated Hospital of Dali University, Kunming, Yunnan, China; 2 Department of Pharmacy, Jinyun People's Hospital, Jinyun, Zhejiang, China; 3 Hospital Administration Office, The Third People’s Hospital of Yunnan Province, The Second Affiliated Hospital of Dali University, Kunming, Yunnan, China

**Keywords:** continuous infusion, critically ill, intermittent infusion, meta-analysis, nephrotoxicity, vancomycin

## Abstract

**Background:**

Vancomycin is a first-line antibiotic for severe gram-positive infections in critically ill patients; however, its narrow therapeutic window and marked interindividual pharmacokinetic variability pose challenges in achieving optimal exposure. This meta-analysis aimed to compare the efficacy and safety of continuous vs. intermittent vancomycin infusion in critically ill patients.

**Methods:**

We systematically searched PubMed, Embase, the Cochrane Central Register of Controlled Trials, and Web of Science. Randomized controlled trials and observational studies were included. Statistical analyses were performed using Stata 19.0, and pooled risk ratios (RR) or mean differences (MD) with 95% confidence intervals (CI) were calculated using random-effects models. Risk of bias was assessed using the Cochrane RoB 2.0 tool for RCTs and the ROBINS-I tool for observational studies; publication bias was evaluated using funnel plots and Egger's test. Sensitivity analyses were performed using the leave-one-out method to assess the robustness of the findings. The certainty of evidence was graded using the GRADE approach.

**Results:**

Twenty-two studies (2,140 patients) were included. Continuous infusion significantly improved serum concentration attainment (RR = 2.23, 95% CI: 1.77–2.80, *P* < 0.001; *I*^2^ = 50.8%) and AUC/MIC attainment (RR = 2.17, 95% CI: 1.60–2.95, *P* < 0.001; *I*^2^ = 39.7%), and significantly reduced the risk of nephrotoxicity (RR = 0.57, 95% CI: 0.46–0.71, *P* < 0.001; *I*^2^ = 0.0%). No significant difference in mortality was observed between the two groups (RR = 1.05, 95% CI: 0.85–1.30, *P* = 0.629; *I*^2^ = 0.0%). Continuous infusion was associated with significantly higher steady-state concentrations (MD = 6.81 mg/L, 95% CI: 3.09–10.54, *P* < 0.001; *I*^2^ = 90.4%) and shorter time to target attainment (MD = −21.64 h, 95% CI: −35.99 to −7.29, *P* = 0.003; *I*^2^ = 84.1%), but no significant difference in AUC/MIC ratio was found (MD = 66.74, 95% CI: −56.20 to 189.67, *P* = 0.287; *I*^2^ = 89.8%). Publication bias was detected for serum concentration attainment (Egger *P* = 0.001); however, the effect estimate remained significant after trim-and-fill correction (adjusted RR = 1.89, 95% CI: 1.38–2.58). The certainty of evidence was moderate for the primary outcomes and low for the secondary outcomes.

**Conclusion:**

Compared with intermittent infusion, continuous vancomycin infusion significantly improves target concentration attainment and reduces nephrotoxicity without increasing mortality. These findings should be interpreted in the context of potential publication bias and between-study heterogeneity.

**Systematic Review Registration:** (https://www.crd.york.ac.uk/PROSPERO/view/CRD420261431488), identifier CRD420261431488.

## 1 Introduction

In critically ill patients, gram-positive bacterial infections remain a leading cause of high mortality, with methicillin-resistant Staphylococcus aureus (MRSA) and coagulase-negative staphylococci (CoNS) being the most frequently isolated pathogens (1, 2). These infections frequently culminate in sepsis, septic shock, and multiorgan failure, underscoring the critical importance of timely and effective antimicrobial therapy in improving patient outcomes (3). Vancomycin, a glycopeptide antibiotic introduced in the 1950s, has long been established as a first-line agent for severe gram-positive infections, and continues to be recommended as the preferred treatment for MRSA infections in major clinical guidelines (4, 5).

However, vancomycin is characterized by a narrow therapeutic window and substantial interindividual pharmacokinetic variability, rendering its efficacy and safety highly dependent on precise dose individualization (6). In critically ill patients, vancomycin pharmacokinetics are particularly unpredictable, owing to alterations in volume of distribution, dynamic renal function, hypoalbuminemia, and concurrent drug therapy (7, 8). Subtherapeutic concentrations may result in treatment failure and foster antimicrobial resistance, whereas supratherapeutic levels substantially increase the risk of nephrotoxicity (9, 10). Consequently, therapeutic drug monitoring (TDM) is regarded as the cornerstone of safe and effective vancomycin therapy (11).

Traditionally, vancomycin is administered via intermittent intravenous infusion (IIV), typically every 6 to 12 h. However, this regimen frequently produces pronounced fluctuations in serum drug concentrations, giving rise to alternating periods of excessively high peak levels and subtherapeutic trough levels (12, 13). As an alternative, continuous intravenous infusion (CIV) delivers the drug at a constant rate over 24 h, which, theoretically, maintains stable serum concentrations, achieves target therapeutic levels more rapidly, and reduces toxicity associated with high peak concentrations (14).

Despite the theoretical advantages of continuous infusion, current clinical guidelines provide inconsistent recommendations regarding the use of vancomycin CIV. For instance, Japanese guidelines advise against the use of continuous or extended infusion of glycopeptides for sepsis treatment (15), whereas others list CIV as an alternative option (4). In recent years, numerous clinical studies have compared the two infusion regimens in critically ill patients, but their conclusions have been inconsistent owing to variations in study design, sample size, and target concentration ranges (16–18). Previous systematic reviews have addressed this question (19, 20); however, they included older studies, omitted recently published RCTs, and did not provide a comprehensive quantitative synthesis of both efficacy and safety outcomes.

Therefore, we conducted an updated systematic review and meta-analysis to comprehensively compare the efficacy and safety of continuous vs. intermittent vancomycin infusion in critically ill patients, aiming to provide more robust evidence to support clinical decision-making.

## 2 Methods

### 2.1 Design

This systematic review and meta-analysis was conducted and reported in accordance with the Cochrane Handbook for Systematic Reviews of Interventions and the Preferred Reporting Items for Systematic Reviews and Meta-Analyses (PRISMA) guidelines (21). The complete PRISMA checklist is available as Supplementary Table 1. The study protocol was prospectively registered with PROSPERO (CRD420261431488), accessible at https://www.crd.york.ac.uk/PROSPERO/view/CRD420261431488.

### 2.2 Data sources and search strategy

We systematically searched PubMed, Embase, the Cochrane Central Register of Controlled Trials (CENTRAL), and Web of Science from inception to June 30, 2026. The search strategy combined controlled vocabulary and free-text keywords (MeSH and Emtree) related to “vancomycin,” “continuous infusion,” and “intermittent infusion” using Boolean operators. No language restrictions were applied. The full search strategy is detailed in Supplementary Table 2. In addition, we manually screened the reference lists of included studies and relevant review articles to identify any further eligible studies.

### 2.3 Eligibility criteria and study selection

Studies were included based on the PICOS framework as follows:

Population: Patients with suspected or confirmed bacterial infection requiring vancomycin therapy, including critically ill patients treated in the intensive care unit (ICU) as well as patients with comparable infection severity in other settings where direct comparative data on infusion modalities were available.
Intervention: CIV of vancomycin.
Comparator: IIV of vancomycin.
Outcomes: The primary outcomes were (1) serum concentration attainment, defined as the proportion of patients achieving the predefined target vancomycin concentration; (2) AUC/MIC attainment, defined as the proportion of patients achieving an AUC/MIC ratio ≥ 400; (3) nephrotoxicity, defined according to KDIGO, AKIN, or RIFLE criteria, or as a ≥50% increase in serum creatinine from baseline; and (4) mortality. Secondary outcomes included steady-state concentration, AUC/MIC ratio, and time to target attainment.
Study design: Randomized controlled trials (RCTs) and non-randomized studies (including prospective and retrospective cohort studies) were eligible for inclusion.

Reviews, case reports, case series, editorials, conference abstracts, and overlapping or duplicate reports were excluded. Two reviewers (YZ and PH) independently screened titles and abstracts to identify potentially eligible studies. Full texts of selected studies were then assessed for final inclusion; disagreements were resolved by discussion or, when necessary, by consultation with a third reviewer (YS).

### 2.4 Data extraction

Data extraction was conducted independently by two reviewers (YZ and LZ) using a standardized data extraction form. The extracted variables included first author, year of publication, country, study design, sample size, age, sex, infection type, causative pathogens, vancomycin dosing regimen, and outcome data. For studies reporting continuous outcomes as median and interquartile range, the mean and standard deviation were estimated using the method described by Wan et al. (22). Disagreements were resolved through discussion or, when necessary, by consulting a third reviewer (RC).

### 2.5 Quality assessment

The risk of bias was assessed independently by two reviewers (YZ and RC). For randomized controlled trials, the Cochrane Risk of Bias 2.0 (RoB 2.0) tool (23) was used to evaluate five domains: (1) the randomization process; (2) deviations from intended interventions; (3) missing outcome data; (4) measurement of the outcome; and (5) selection of the reported result. Each domain was rated as “low risk,” “some concerns,” or “high risk.” For non-randomized studies, the Risk of Bias in Non-randomized Studies of Interventions (ROBINS-I) tool (24) was used to assess seven domains: (1) confounding; (2) selection of participants; (3) classification of interventions; (4) deviations from intended interventions; (5) missing data; (6) measurement of outcomes; and (7) selection of the reported result. Each domain was rated as “low,” “moderate,” “serious,” or “critical” risk of bias, with an overall judgment determined accordingly. Disagreements were resolved through consensus or by consultation with a third reviewer (YS).

### 2.6 Statistical analysis

All statistical analyses were conducted using Stata version 19.0 (StataCorp, College Station, TX, USA). For dichotomous outcomes, pooled risk ratios (RRs) with 95% confidence intervals (CIs) were calculated using the Mantel-Haenszel method. For continuous outcomes, pooled mean differences (MDs) with 95% CIs were calculated using the inverse-variance method. Heterogeneity was assessed using the *I*^2^ statistic and Cochran's *Q* test. An *I*^2^ > 50% was considered indicative of substantial heterogeneity, in which case a random-effects model was applied; otherwise, a fixed-effects model was used. A *P*-value < 0.10 for the *Q* test was considered statistically significant for heterogeneity.

To explore potential sources of heterogeneity, pre-specified subgroup analyses were performed for the serum concentration attainment according to study design (RCTs vs. non-randomized studies) and patient population (children vs. adults). Subgroup differences were assessed using interaction tests within the random-effects model framework; an interaction *P*-value < 0.10 was considered statistically significant.

For outcomes with sufficient data (≥10 studies), publication bias was assessed by visual inspection of funnel plots and by Egger's regression asymmetry test. A *P*-value < 0.05 was considered indicative of statistically significant publication bias; when detected, the trim-and-fill method was applied to adjust for it. Sensitivity analyses were performed by sequentially excluding individual studies (leave-one-out method) to evaluate the robustness of the pooled estimates. For studies rated as having serious risk of bias, we performed an additional sensitivity analysis excluding these studies and repeating the meta-analyses for all outcomes to assess the robustness of our findings.

### 2.7 Assessment of evidence quality

The certainty of evidence for each outcome was assessed using the GRADE (Grading of Recommendations, Assessment, Development, and Evaluations) framework. The assessment considered five domains: risk of bias, inconsistency, indirectness, imprecision, and publication bias. Evidence from RCTs started as high certainty but could be downgraded based on serious or very serious concerns in any of these domains. Evidence from observational studies was initially assigned low certainty and could be further downgraded if limitations were identified. The GRADE assessment was performed independently by two reviewers, with disagreements resolved through discussion.

## 3 Results

### 3.1 Study selection

The study selection process is presented in the PRISMA flow diagram (Figure 1). A systematic search of PubMed, Embase, Cochrane Library, and Web of Science yielded 1,782 records. After removing 668 duplicates, 1,114 records were screened by title and abstract, of which 1,010 were excluded. The remaining 104 full-text articles were assessed for eligibility, and 82 were excluded based on the predefined inclusion and exclusion criteria. Ultimately, 22 studies were included in the meta-analysis.

**Figure 1:**
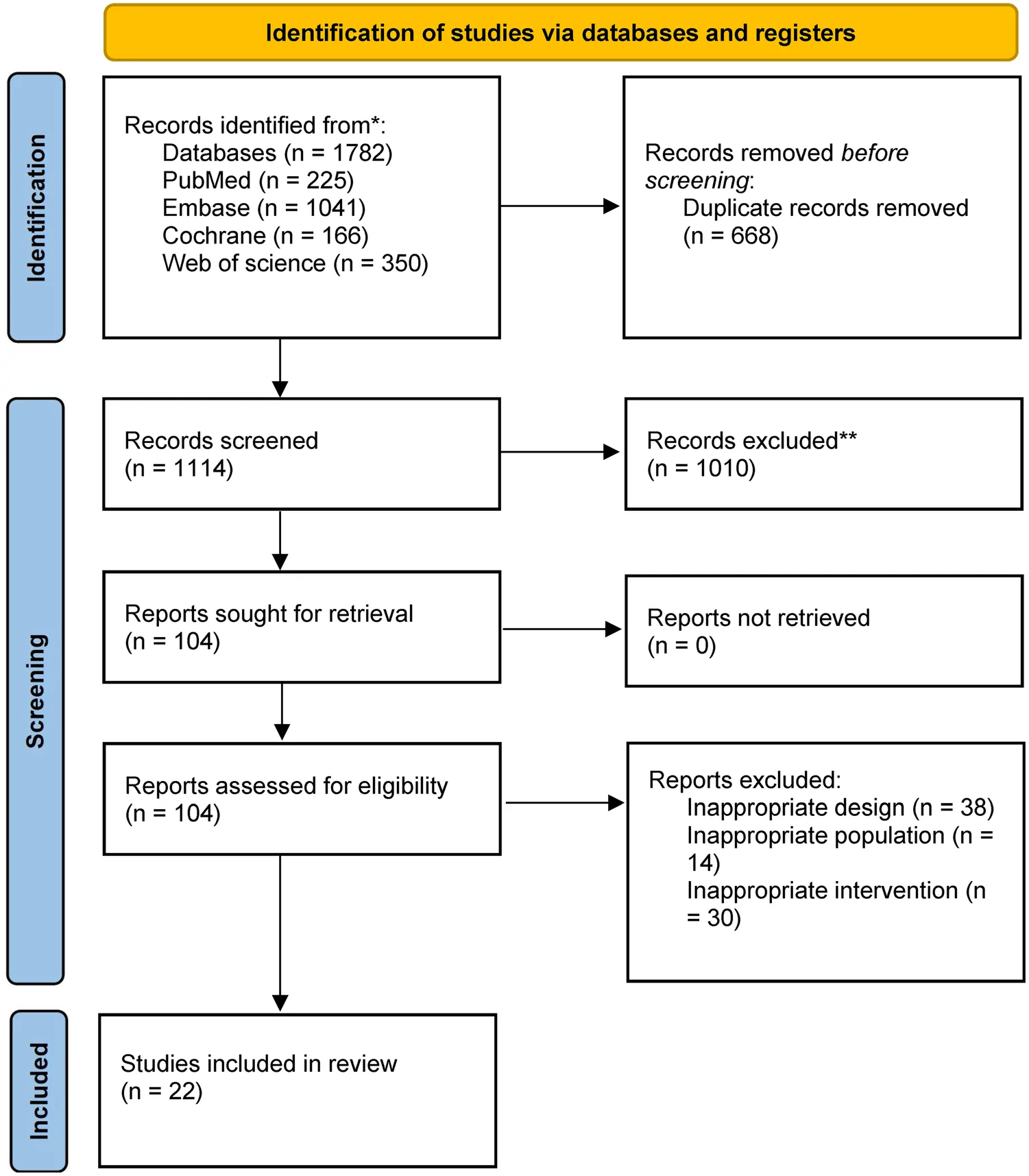
PRISMA flow diagram of study selection process.

### 3.2 Baseline characteristics of the included studies

The baseline characteristics of each included study are summarized in Table 1. A total of 22 studies were included, comprising 5RCTs and 17 non-randomized studies. The studies were published between 2001 and 2026 and were conducted across 11 countries, with the majority from the United States, France, China, Australia, and Germany. The total sample size across all studies was 2,140 participants, with 1,162 in the CIV group and 978 in theIIV group. Study populations varied considerably: 17 studies enrolled adult patients, while 5 focused on pediatric or neonatal populations. The mean age of adult patients ranged from 38.3 to 71.1 years. In most studies, the proportion of male participants ranged from 50.0% to 94.5%. The most common infection types were sepsis/bacteremia, pneumonia, and skin and soft tissue infections. The predominant pathogens were Staphylococcus aureus including MRSA and CoNS. MIC data were available in a subset of studies. MIC distributions differed substantially across species, with reported MIC values ranging from ≤0.5 mg/L to 4 mg/L across different pathogens. Vancomycin dosing regimens varied across studies: CIV regimens typically consisted of a loading dose (15–25 mg/kg) followed by continuous maintenance infusion (20–60 mg/kg/day), whereas IIV regimens involved intermittent administration every 6 to 24 h (7.5–60 mg/kg/day).

**Table 1:** Baseline characteristics of the included studies.

Study	Country	Design	Intervention	Sample size, N(CIV/IIV)	Age, Mean ± SD	Male, n (%)	Infection type	Microbiology, %		MIC, *n*/Mean ± SD	
								CIV	IIV	CIV	IIV
Wu et al., 2026 (25)	China	RCT	CIV: Loading dose 20 mg/kg, followed by continuous infusion 20 mg/kg/day (total 40 mg/kg/day); IIV: 10 mg/kg per dose q6 h (total 40 mg/kg/day)	80 (40/40)	1.89 ± 3.19 yr	54 (67.5%)	Sepsis, pneumonia, purulent meningitis, SSTI, osteoarthritis	Staphylococcus aureus (10%), Streptococcus pneumoniae (12.5%), GBS (5%), Enterococcus (5%), CoNS (5%), MRSA (5%)	Staphylococcus aureus (37.5%), Streptococcus pneumoniae (10%), GBS (5%), Enterococcus (2.5%), CoNS (5%), MRSA (2.5%)	① MIC ≤ 0.5 mg/L(4)② MIC = 1 mg/L(13)	① MIC ≤ 0.5 mg/L(15)② MIC = 1 mg/L(9)③ MIC = 2 mg/L(1)
Gérard et al., 2024 (27)	France	Retrospective cohort (PSM)	CIV: Model-based loading and maintenance doses; IIV: 15 mg/kg per dose	76 (29/47)	27.2 ± 3.6 wk (GA)	NR	Late-onset neonatal sepsis (CoNS BSI)	Staphylococcus epidermidis (55.2%), Staphylococcus haemolyticus (20.7%), Staphylococcus capitis (20.7%), Staphylococcus hominis (3.4%)	Staphylococcus epidermidis (38.3%), Staphylococcus haemolyticus (42.5%), Staphylococcus capitis (12.8%), Staphylococcus hominis (2.1%), Staphylococcus warneri (4.3%)	1.1 ± 0.45	1.39 ± 0.61
Roshan N.S. et al., 2024 (17)	Iran	RCT	CIV: Loading dose 15 mg/kg, followed by continuous infusion 60 mg/kg/day (total 60 mg/kg/day); IIV: 15 mg/kg per dose q6 h (total 60 mg/kg/day)	68 (33/35)	3.25 ± 3.65 yr	43 (63.2%)	Suspected/proven MRSA infection	NR	NR	NR	NR
Xu et al., 2022 (42)	China	Prospective cohort	CIV: Loading dose 20–25 mg/kg, followed by EFR-based continuous infusion; IIV: 7.5–15 mg/kg per dose q12 h (total 15–30 mg/kg/day)	51 (23/28)	71.06 ± 16.96 yr	36 (70.6%)	Lung, bloodstream, urinary tract, intra-abdominal, SSTI, endocarditis	MRSA (61.1%), Enterococcus faecium (22.2%), Staphylococcus epidermidis (11.1%), Staphylococcus haemolyticus (5.6%)	MRSA (50%), Enterococcus faecium (27.8%), Staphylococcus epidermidis (5.6%), Staphylococcus haemolyticus (11.1%), Staphylococcus hominis (5.6%)	①MIC ≤ 1 mg/L(16)②MIC = 2 mg/L(2)	①MIC ≤ 1 mg/L(*n* = 13)②MIC = 2 mg/L(*n* = 3)
Schlobohm et al., 2021 (38)	USA	Retrospective cohort	CIV: Pea nomogram-based dosing, CrCl-guided; IIV: CrCl-based nomogram dosing	60 (30/30)	45.9 ± 16.6 yr	51 (85.0%)	Burn injury	S. aureus was the most common pathogen	S. aureus was the most common pathogen	NR	NR
Shakeraneh et al., 2020 (30)	USA	Retrospective cohort (PSM)	CIV: cont.; IIV: intermittent	148 (74/74)	53.7 ± 14.1 yr	82 (55.4%)	Bone and joint, bloodstream, CNS, SSTI, endocarditis	NR	NR	NR	NR
Bissell et al., 2020 (32)	USA	Retrospective cohort	CIV: Loading dose 20 mg/kg, followed by continuous infusion 30 mg/kg/day (total 30 mg/kg/day); IIV: Interval q8 h/q12 h/q24 h based on renal function	150 (75/75)	47.5 ± 17.2 yr	120 (80.0%)	Pneumonia, meningitis, empiric, bacteremia, SSTI, abdominal	MRSA (16%), MSSA (16%), Streptococcus (4%), Other Staphylococcus (9.3%), Enterococcus (4%), gram-negative rods (30.7%)	MRSA (26.7%), MSSA (5.3%), Streptococcus (2.7%), Other Staphylococcus (12%), Enterococcus (5.3%), gram-negative rods (29.3%), Fungal(1.3%)	NR	NR
Gwee et al., 2019 (16)	Australia	RCT	CIV: Loading dose 15 mg/kg, followed by CGA- and Cr-based maintenance (20–50 mg/kg/day); IIV: 15 mg/kg per dose q24 h/q12 h/q8 h/q6 h (CGA-based)	104 (53/51)	34.2 ± 4.8 wk (GA)	52 (50.0%)	Suspected sepsis, preceding positive blood culture result, SSTI, surgical prophylaxis, gastrointestinal infection, respiratory tract infection, endocarditis, septic ileus, urinary tract infection	CoNS (13%), Enterococcus spp (2%), MSSA (2%), GBS (2%), gram-negative organism (2%), 2 different CONS bacteria (2%)	CoNS (15%), Enterococcus spp (2%), Streptococcus mitis group (2%),gram-negative organism (2%), 2 different CONS bacteria (4%)	①MIC ≤ 0.5 mg/L(2)② MIC = 1 mg/L(1)③MIC = 2 mg/L(8)④MIC = 4 mg/L(6)	
Duszynska et al., 2016 (35)	Poland	Prospective cohort	CIV: Loading dose 0.5 g, followed by continuous infusion 30 mg/kg/day; IIV: Loading dose 25 mg/kg, followed by nomogram-guided maintenance	42 (21/21)	58.0 ± 14.5 yr	35 (83.3%)	Peritonitis, CLA-BSI, SSTI, CNS, HAP/VAP	CoNS (71%), Enterococcus faecium (24%), Streptococcus pneumoniae (5%)	CoNS (62%), Enterococcus faecium (38%)	2 ± 0.25	2 ± 0.38
Maarseveen et al., 2016 (40)	Netherlands	Retrospective cohort	CIV: Loading dose 1,000 mg, followed by GFR-based maintenance infusion; IIV: 15 mg/kg per dose q12 h;	71 (44/27)	67.1 ± 12.4 yr	47 (66.2%)	Sepsis, pneumonia, endocarditis	NR	NR	NR	NR
Demirel et al., 2015 (34)	Turkey	Retrospective cohort	CIV: Loading dose 10 mg/kg, followed by continuous infusion with total daily dose calculated from intermittent dosing per Neofax; IIV: Per Neofax recommendations	77 (36/41)	29.0 ± 2.9 wk (GA)	47 (61.0%)	Bacteremia, meningitis, VAP, UTI	MR staphylococcus sp.(19.4%), MS staphylococcus sp.(5.5%)	MR staphylococcus sp.(29.2%), MS staphylococcus sp.(2.4%)	NR	NR
Hong et al., 2015 (28)	USA	Retrospective cohort	CIV: Loading dose 20 mg/kg, followed by maintenance infusion 15–40 mg/kg/day; IIV: Approximately 15 mg/kg per dose, interval q8 h–q24 h	130 (65/65)	56.2 ± 15.2 yr	71 (54.6%)	Pneumonia, Meningitis, Empiric, Epidural abscess, Bacteremia	MSSA (66.7%), Enterococcus (6.7%), CoNS(6.7%), MRSA（6.7%)，MRSE(13.3%)	MSSA (42.9%), Enterococcus (14.3%), CoNS(14.3%), MRSA（21.4%), MSSA and Enterococcus (7.1%)	①MIC = 0.5 mg/L(2)②MIC = 1 mg/L(13)	①MIC = 0.5 mg/L(2)②MIC = 1 mg/L(10)③ MIC = 2 mg/L(2)
Lin et al., 2015 (14)	USA	Retrospective cohort	CIV: Loading dose 25 mg/kg, followed by continuous infusion 1,500 mg/day; IIV: Initial dose 1,000–2,000 mg/day (once daily)	59 (14/45)	63.4 ± 15.0 yr	42 (71.2%)	NR	NR	NR	NR	NR
Tafelski et al., 2015 (39)	Germany	Prospective cohort	CIV: Loading dose 1 g, followed by continuous infusion 0.5–2 g/day; IIV: 500 mg per dose q6 h or 1 g per dose q12 h	125 (76/49)	61.3 ± 17.0 yr	79 (63.2%)	Pneumonia, endocarditis, urogenital, CNS, Aabdomen, catheter-related, bones and joints, soft tissues	NR	NR	NR	NR
Blot et al., 2014 (33)	Australia	Prospective cohort	CIV: No standardized protocol; IIV: No standardized protocol	42 (24/18)	56.8 ± 15.6 yr	27 (64.3%)	NR	NR	NR	MIC = 1 mg/L(2)	
Schmelzer et al., 2013 (18)	USA	RCT	CIV: Loading dose 20 mg/kg, followed by maintenance infusion 0.9–2.4 mg/kg/h; IIV: 15 mg/kg per dose q12 h (total 30 mg/kg/day)	55 (28/27)	40.8 ± 17.2 yr	52 (94.5%)	Trauma ICU with suspected VAP	MRSA(<10%)	MRSA(<10%)	NR	NR
Saugel et al., 2013 (37)	Germany	Retrospective cohort	CIV: Loading dose 1,000/1,250 mg, followed by continuous infusion 60 mg/h (normal renal function) or 40 mg/h (renal impairment); IIV: 1,000–2,000 mg/d	239 (164/75)	63.8 ± 13.7 yr	150 (62.8%)	Suspected or diagnosed infections due to gram-positive bacteria	NR	NR	NR	NR
Akers et al., 2012 (31)	USA	Retrospective cohort	CIV: 3 g/day continuous infusion; IIV: 1 g per dose q8 h	171 (90/81)	38.3 ± 18.6 yr	155 (90.6%)	gram-positive Bacteremia, Sepsis without proven gram-positive bacteremia, Pneumonia	Staphylococcus aureus (59.4%), MRSA (25.5%), Enterococcus faecium (12.3%), Enterococcus faecalis (4.7%), Streptococcus pneumoniae (3.8%), Streptococcus viridans (1.9%), Streptococcus agalactiae (1.9%), Nonenterococcal Group D Streptococci (1.9%), Streptococcus mutans (0.9%)		MIC_50_ and MIC_90_ to be 0.75 and 1.5 *μ*g/mL	
Ingram et al., 2009 (29)	Singapore	Retrospective cohort (PSM)	CIV: 500 mg/h continuous infusion via elastomeric infuser; IIV: Clinician-directed intermittent dosing	80 (40/40)	52.9 ± 17.8 yr	49 (61.3%)	Osteoarticular infection, SSTI	MRSA (67.5%), CoNS (12.5%)	MRSA (67.5%), CoNS (7.5%), Enterococcus sp. (10%)	NR	NR
Hutschala et al., 2009 (36)	Austria	Retrospective cohort	CIV: Loading dose 20 mg/kg, followed by continuous infusion 0.025 mg/kg/min; IIV: Loading dose 20 mg/kg, followed by intermittent infusion	149 (119/30)	59.0 ± 14.0 yr	93 (62.4%)	Wound infection, sternal infection, mediastinitis, catheter-related infection, hospital-acquired pneumonia	Staphylococcus aureus (22%)	Staphylococcus aureus (26%)	NR	NR
Vuagnat et al., 2004 (41)	France	Prospective cohort	CIV: Loading dose 20 mg/kg, followed by continuous infusion 40 mg/kg/day; IIV: Loading dose 20 mg/kg, followed by 20 mg/kg per dose q12 h	44 (23/21)	56.7 yr	NR	Osteomyelitis	MRSA (70.5%), MRCNS (28%)		1.9 ± 0.8	1.8 ± 0.6
Wysocki et al., 2001 (26)	France	RCT	CIV: Loading dose 15 mg/kg, followed by continuous infusion 30 mg/kg/day; IIV: 15 mg/kg per dose q12 h	119 (61/58)	63.0 ± 14.5 yr	77 (64.7%)	Endocarditis, septicemia, catheter-related infection, pneumonia, mediastinitis, meningitis	Staphylococcus aureus (80%), CoNS (20%)	Staphylococcus aureus (82%), CoNS (18%)	①MIC = 0.5 mg/L(3)②MIC = 1 mg/L(8)③ MIC = 2 mg/mL(11)	①MIC = 1 mg/L(5)② MIC = 2 mg/mL(13)

N, total sample size; CIV, continuous intravenous infusion; IIV, intermittent intravenous infusion; yr, years; wk, weeks. NR, not reported; n, sample size for each group. GA, gestational age; RCT, randomized controlled trial; PSM, propensity score matching; CoNS, coagulase-negative staphylococci; MRSA, methicillin-resistant Staphylococcus aureus; MRCNS, methicillin-resistant coagulase-negative staphylococci; MSSA, methicillin-susceptible Staphylococcus aureus; VAP, ventilator-associated pneumonia; SSTI, skin and soft tissue infection; BSI, bloodstream infection; CNS, central nervous system; EFR, effluent flow rate; GA, gestational age; ICU, intensive care unit; GBS, Group B Streptococcus; AUC, area under the concentration-time curve; MIC, minimum inhibitory concentration.

### 3.3 Assessment of risk of bias

Among the five RCTs, four were rated as having a low overall risk of bias (16, 17, 25, 26). One study (18) was judged as having some concerns, primarily due to a “some concerns” rating in the domain of deviations from intended interventions (D2). The summary of the RoB 2.0 assessments is presented in Figure 2.

**Figure 2:**
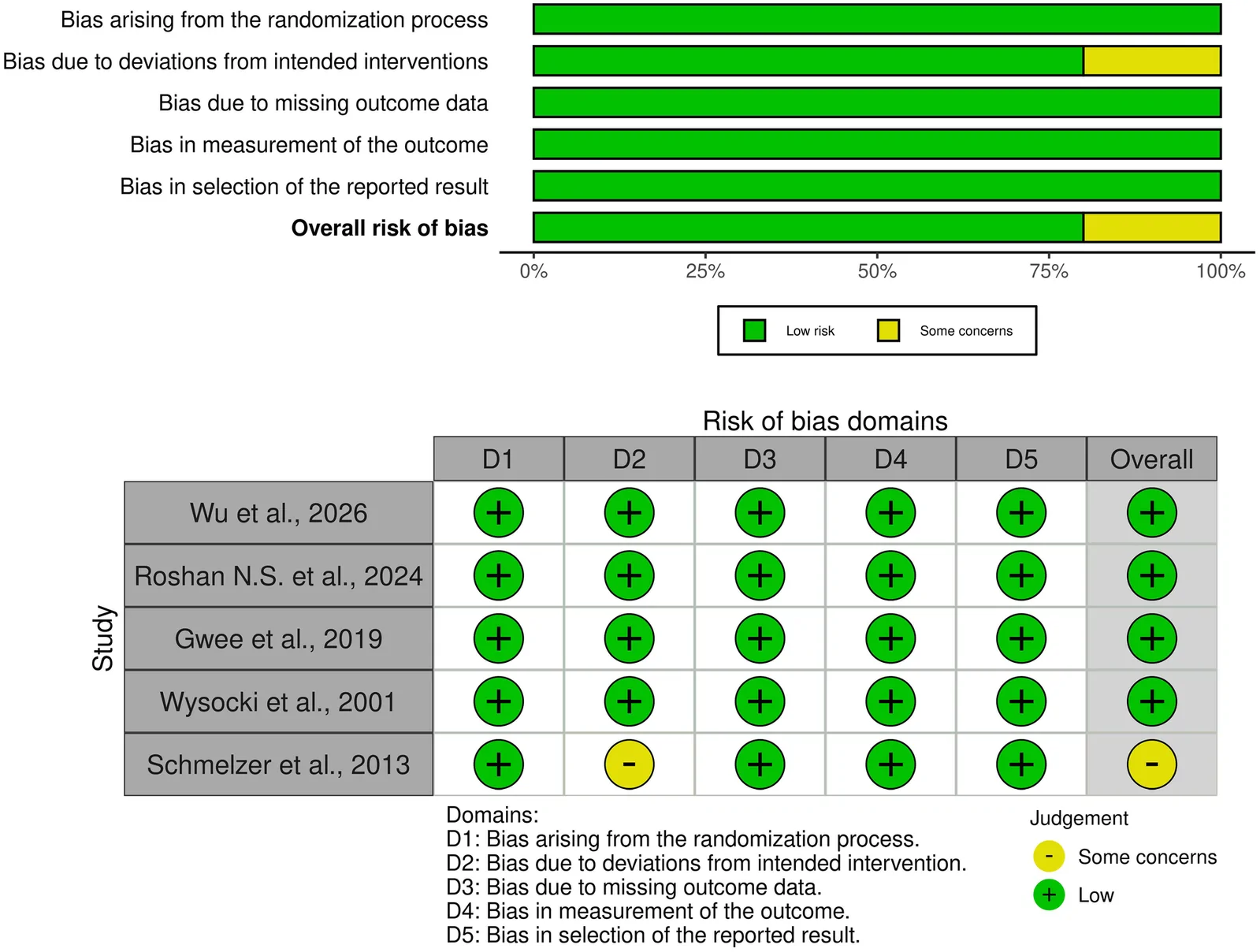
Risk of bias assessment of included randomized controlled trials using the cochrane RoB 2.0 tool.

Among the 17 non-randomized studies, four were rated as having a moderate overall risk of bias (27–30). All four were propensity score-matched cohort studies that adequately adjusted for key confounders. The remaining 13 non-randomized studies (14, 31–42) were rated as having a serious overall risk of bias, primarily due to confounding and selection of participants, which largely stemmed from retrospective designs, lack of adjustment for important confounders, or the use of historical controls (Supplementary Figure 1).

### 3.4 Outcomes and effect sizes

#### 3.4.1 Serum concentration attainment

Thirteen studies (14, 16, 18, 25, 27, 28, 32–35, 38, 40, 42), comprising 997 participants, reported serum concentration attainment. A random-effects meta-analysis showed that the attainment rate was significantly higher in the continuous infusion group than in the intermittent infusion group (RR = 2.23, 95% CI: 1.77–2.80, *P* < 0.001), with moderate heterogeneity among studies (*I*^2^ = 50.8%, *P* = 0.018). The corresponding forest plot is presented in Figure 3A.

**Figure 3:**
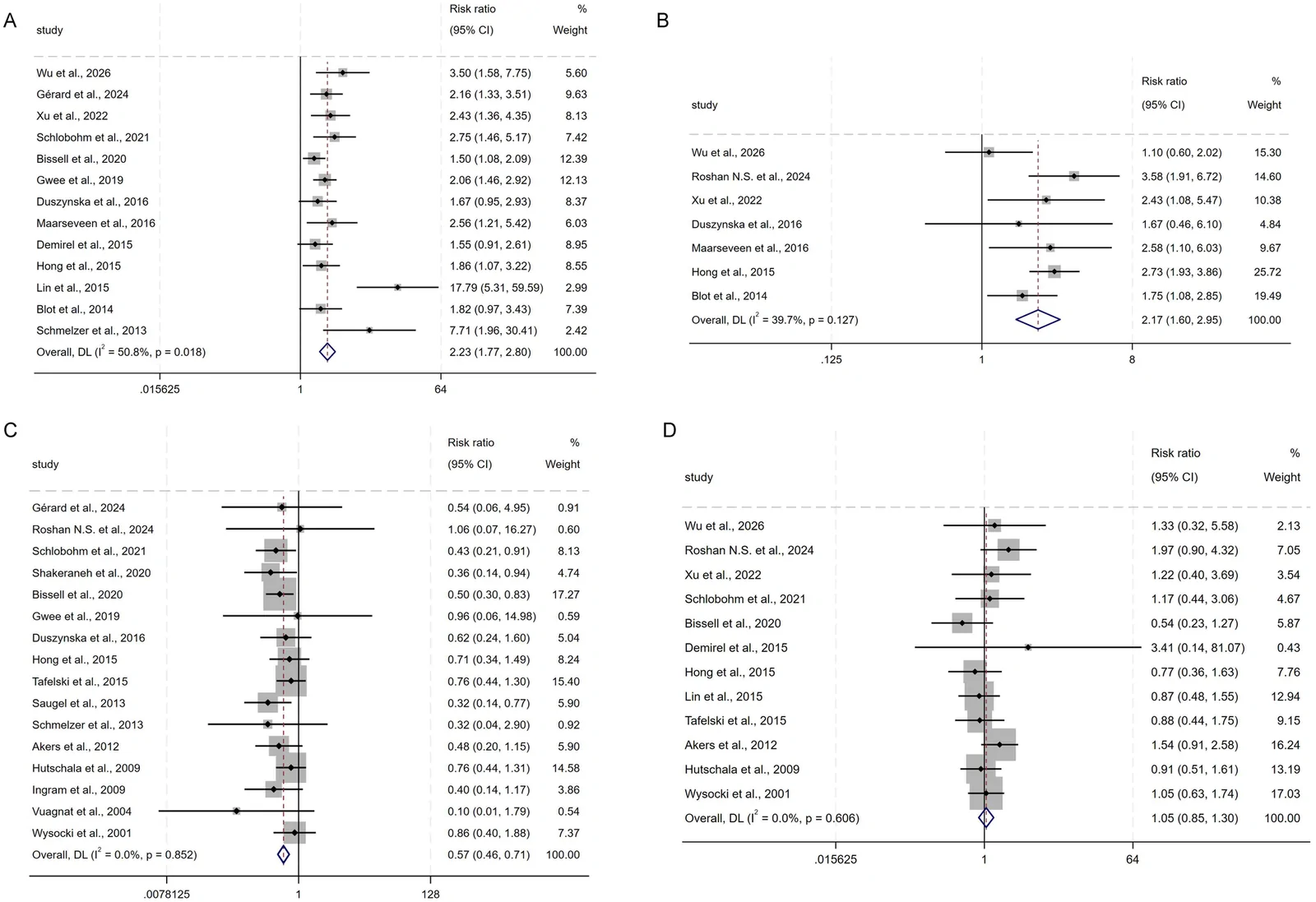
Forest plots of binary outcomes comparing continuous vs. intermittent vancomycin infusion. **(A)** Serum concentration attainment; **(B)** AUC/MIC attainment; **(C)** Nephrotoxicity; **(D)** Mortality.

Subgroup analysis by study design yielded consistent results between RCTs (RR = 3.02, 95% CI: 1.59–5.75, *I*^2^ = 54.4%, *P* = 0.112) and non-randomized studies (RR = 2.11, 95% CI: 1.62–2.74, *I*^2^ = 51.9%, *P* = 0.028), with no significant subgroup difference (interaction *P* = 0.310) (Supplementary Figure 2). Similarly, subgroup analyses by patient population yielded consistent findings for children (RR = 2.06, 95% CI: 1.63–2.61, *I*^2^ = 0.0%, *P* = 0.408) and adults (RR = 2.42, 95% CI: 1.70–3.43, *I*^2^ = 62.8%, *P* = 0.006), with no significant subgroup difference (interaction *P* = 0.460) (Supplementary Figure 3). These findings suggest that neither study design nor patient population was a major source of heterogeneity, and that the benefit of continuous infusion in improving serum concentration attainment was consistent across different study designs and age groups.

#### 3.4.2 AUC/MIC attainment

Seven studies (17, 25, 28, 33, 35, 40, 42), comprising 446 participants, reported AUC/MIC attainment. AUC estimation methods included Bayesian estimation (17, 25, 40), Css × 24 for CIV (42), two-compartment model (35), one-compartment equations (28), and trapezoidal (33). A random-effects meta-analysis showed that the attainment rate was significantly higher in the continuous infusion group than in the intermittent infusion group (RR = 2.17, 95% CI: 1.60–2.95, *P* < 0.001), with low heterogeneity among studies (*I*^2^ = 39.7%, *P* = 0.127) (Figure 3B).

#### 3.4.3 Nephrotoxicity

Sixteen studies (16–18, 26–32, 35–39, 41), comprising 1,663 participants, reported nephrotoxicity outcomes. A random-effects meta-analysis showed that the risk of nephrotoxicity was significantly lower in the continuous infusion group than in the intermittent infusion group (RR = 0.57, 95% CI: 0.46–0.71, *P* < 0.001), with no heterogeneity among studies (*I*^2^ = 0.0%, *P* = 0.852) (Figure 3C).

#### 3.4.4 Mortality

Twelve studies (14, 17, 25, 26, 28, 31, 32, 34, 36, 38, 39, 42), comprising 1,239 participants, reported mortality outcomes. A random-effects meta-analysis showed no statistically significant difference in mortality between the continuous infusion and intermittent infusion groups (RR = 1.05, 95% CI: 0.85–1.30, *P* = 0.629), with no heterogeneity among studies (*I*^2^ = 0.0%, *P* = 0.606) (Figure 3D).

#### 3.4.5 Steady-state concentration

Six studies (14, 17, 18, 25, 41, 42), comprising 395 participants, reported steady-state vancomycin concentrations. A random-effects meta-analysis showed that steady-state concentrations were significantly higher in the continuous infusion group than in the intermittent infusion group (MD = 6.81 mg/L, 95% CI: 3.09–10.54, *P* < 0.001), with high heterogeneity among studies (*I*^2^ = 90.4%, *P* < 0.001) (Figure 4A).

**Figure 4:**
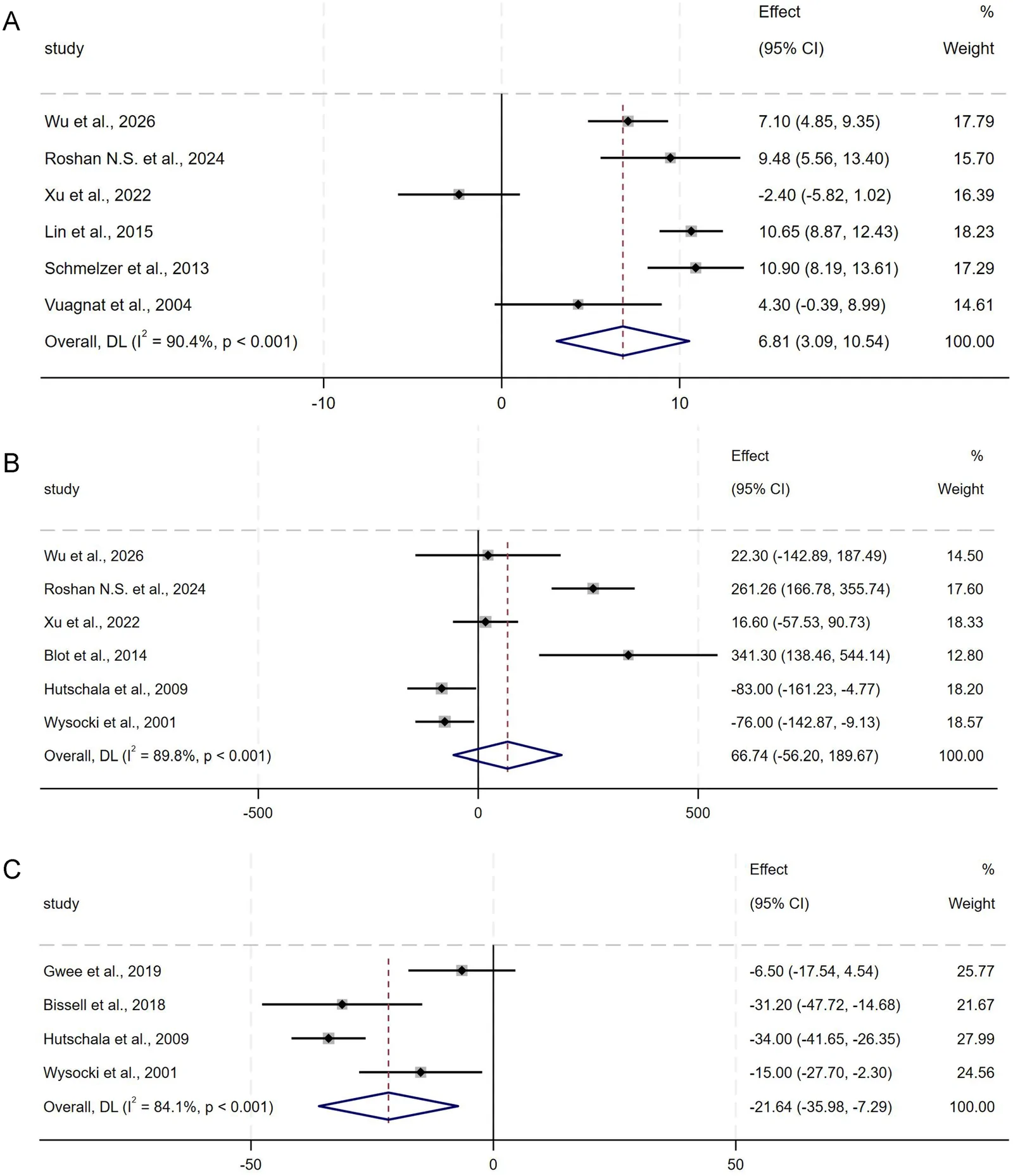
Forest plots of continuous outcomes comparing continuous vs. intermittent vancomycin infusion. **(A)** Steady-state concentration (mg/L); **(B)** AUC/MIC ratio; **(C)** Time to target attainment (hours).

#### 3.4.6 AUC/MIC ratio

Six studies (17, 25, 26, 33, 36, 42), comprising 509 participants, reported the AUC/MIC ratio as a continuous variable. AUC estimation methods included Bayesian estimation (17, 25), Css × 24 for CIV (42), and trapezoidal (26, 33, 36). A random-effects meta-analysis showed that the AUC/MIC ratio did not differ significantly between the continuous infusion and intermittent infusion groups (MD = 66.74, 95% CI: −56.20 to 189.67, *P* = 0.287), with high heterogeneity among studies (*I*^2^ = 89.8%, *P* < 0.001) (Figure 4B).

#### 3.4.7 Time to target attainment

Four studies (16, 26, 32, 36), comprising 522 participants, reported the time to target concentration attainment. A random-effects meta-analysis showed that the time to reach therapeutic concentrations was significantly shorter in the continuous infusion group than in the intermittent infusion group (MD = −21.64 h, 95% CI: −35.99 to −7.29, *P* = 0.003), with high heterogeneity among studies (*I*^2^ = 84.1%, *P* < 0.001) (Figure 4C).

### 3.5 Publication bias

The funnel plot for serum concentration attainment showed asymmetry, and the Egger test confirmed the presence of publication bias (*P* = 0.001). After imputing four studies on the left side using the trim-and-fill method, the adjusted RR remained statistically significant (unadjusted RR = 2.13, 95% CI: 1.77–2.56; adjusted RR = 1.89, 95% CI: 1.38–2.58), indicating that the findings were robust (Figures 5A,B). The funnel plots for nephrotoxicity (*P* = 0.235) and mortality (*P* = 0.693) appeared largely symmetrical, and the Egger tests did not suggest publication bias for either outcome (Figures 5C,D). For outcomes with fewer than 10 included studies (AUC/MIC attainment, steady-state concentration, AUC/MIC ratio, and time to target attainment), funnel plot asymmetry tests have limited power, and Egger's test was therefore not performed.

**Figure 5:**
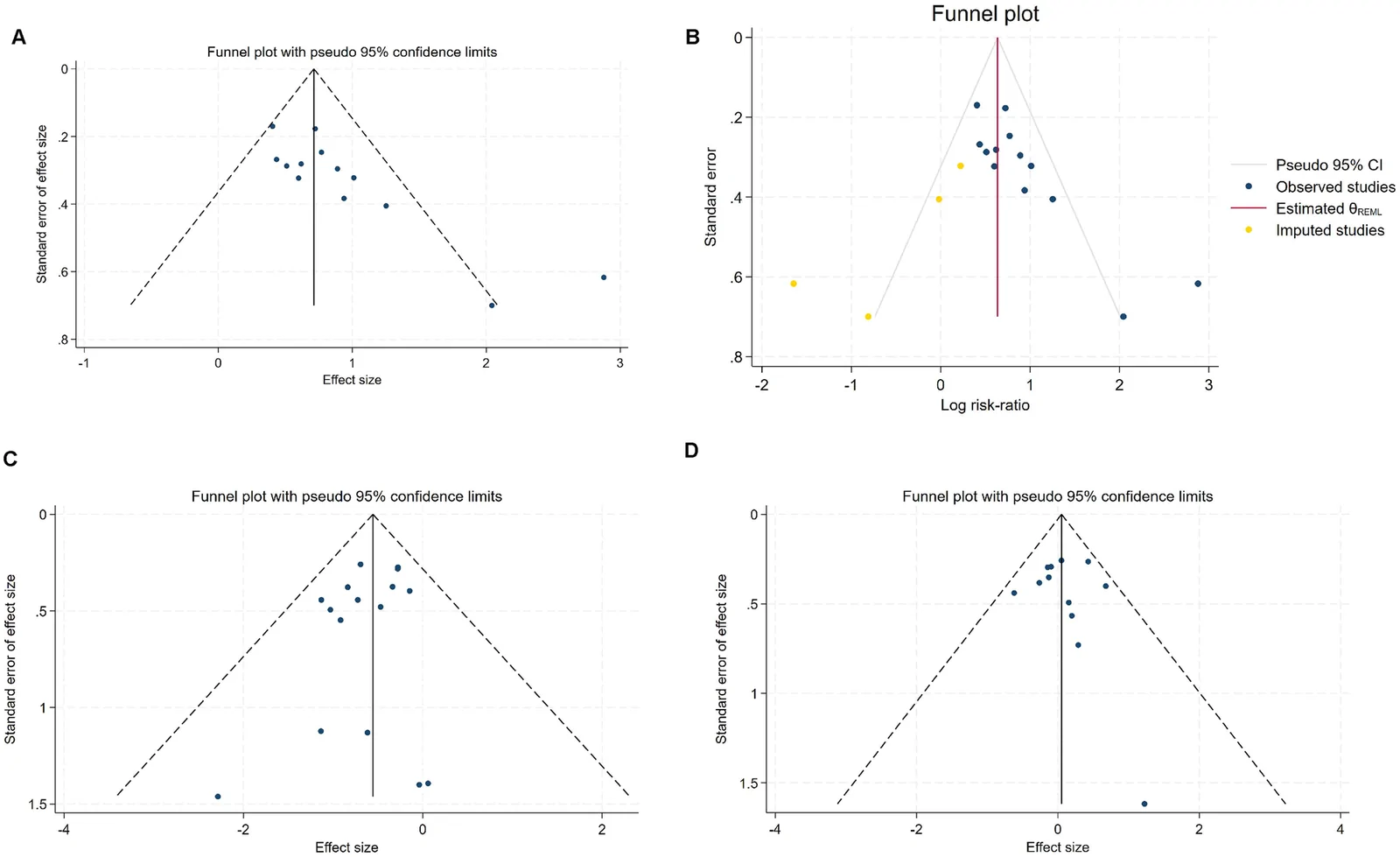
Funnel plots and trim-and-fill analysis for publication bias assessment. **(A)** Funnel plot for serum concentration attainment; **(B)** Trim-and-fill plot for serum concentration attainment (four imputed studies on the left side); **(C)** Funnel plot for nephrotoxicity; **(D)** Funnel plot for mortality.

### 3.6 Sensitivity analysis

Leave-one-out sensitivity analyses showed that the direction of the pooled effect estimates for all outcomes remained unchanged after sequentially excluding each individual study. Serum concentration attainment, AUC/MIC attainment, nephrotoxicity, and time to target attainment remained statistically significant, whereas mortality remained non-significant. For steady-state concentration, exclusion of Xu et al. (42) increased the pooled mean difference to 8.87 mg/L (95% CI: 6.74–11.00) and substantially narrowed the confidence interval, suggesting that this study may be the primary source of heterogeneity. For the AUC/MIC ratio, exclusion of either Roshan N.S. et al. (17) or Blot et al. (33) attenuated the pooled effect size, but the null finding remained unchanged. The corresponding leave-one-out forest plots are presented in Supplementary Figures 4, 5. We also performed a sensitivity analysis excluding the 13 non-randomized studies rated as having serious risk of bias. These sensitivity analyses confirmed that the exclusion of studies with serious risk of bias did not materially alter the direction or statistical significance of any pooled estimate, supporting the robustness of our primary findings (Supplementary Figures 6, 7).

### 3.7 Assessment of the certainty of the evidence

The GRADE assessment of the evidence is summarized in Supplementary Table 3. Serum concentration attainment, AUC/MIC attainment, nephrotoxicity, and mortality were rated as having moderate certainty of evidence, while steady-state concentration, AUC/MIC ratio, and time to target attainment were rated as having low certainty of evidence.

## 4 Discussion

This systematic review and meta-analysis, comprising 22 studies with a total of 2,140 patients, systematically compared the efficacy and safety of continuous vs. intermittent vancomycin infusion in critically ill patients. The key findings demonstrate that continuous infusion significantly improved serum concentration and AUC/MIC attainment, reduced the risk of nephrotoxicity, and shortened the time to target attainment, without a significant difference in mortality between the two groups. These findings were generally consistent across subgroup and sensitivity analyses, although the certainty of evidence varied across outcomes.

Continuous infusion was significantly superior to intermittent infusion in improving serum concentration attainment (RR = 2.23, 95% CI: 1.77–2.80, *P* < 0.001), and this finding remained consistent across RCTs, non-randomized studies, and different patient populations. By maintaining stable serum drug concentrations and avoiding the “peak-and-trough” fluctuations characteristic of intermittent infusion, continuous infusion more reliably sustains levels within the therapeutic window (43). Moreover, it significantly shortens the time to target attainment, which is particularly critical for patients with severe infections such as sepsis, where delayed achievement of therapeutic concentrations is a well-established contributor to adverse outcomes (44).

For AUC/MIC, the attainment rate was significantly higher with continuous infusion (RR = 2.17, 95% CI: 1.60–2.95, *P* < 0.001), consistent with current guidelines that recommend AUC/MIC-guided vancomycin monitoring (4). However, when the AUC/MIC ratio was analyzed as a continuous variable, the difference between groups was not statistically significant (MD = 66.74, 95% CI: −56.20 to 189.67, *P* = 0.287), with substantial heterogeneity. This apparent discrepancy may be explained by the fact that the binary attainment rate provides a clinically meaningful threshold (≥400), whereas the continuous ratio is more susceptible to extreme values and inter-study differences in MIC determination methods.

Current guidelines no longer recommend relying solely on vancomycin trough concentrations for therapeutic drug monitoring, as they represent a single time-point exposure measure at the end of the dosing interval and are derived from the minimum daily area under the curve (AUC) value. Based on a minimum inhibitory concentration (MIC) of 1 mg/L, the target 24 h AUC/MIC ratio range is 400–600 h × mg L⁻¹. This PK/PD index is now recommended for dose guidance to minimize the risk of overexposure and adverse events. Notably, however, PK/PD targets for continuous infusion have not yet been fully validated, as the majority of evidence supporting an AUC/MIC > 400 as the best predictor of favorable clinical outcomes originates from studies using intermittent vancomycin infusion (45).

This meta-analysis demonstrated that continuous infusion significantly reduced the risk of nephrotoxicity (RR = 0.57, 95% CI: 0.46–0.71, *P* < 0.001), with no heterogeneity across the 16 included studies (*I*^2^ = 0.0%). Although continuous infusion was associated with higher steady-state concentrations (MD = 6.81 mg/L), these concentrations are mechanistically distinct from transient peak concentrations. Notably, despite the higher steady-state concentrations in the CIV group, the pooled continuous AUC/MIC ratio showed no statistically significant difference between the two groups, suggesting that overall systemic vancomycin AUC exposure did not differ significantly between the two dosing strategies. Mechanistically, vancomycin accumulates in proximal tubular cells, triggering oxidative stress and apoptosis, with the degree of injury correlating with AUC and higher maximum concentrations (46). Intermittent infusion produces pronounced peak-and-trough fluctuations, exposing renal tubules to repeated high-concentration spikes that may overwhelm cellular defense mechanisms. In contrast, continuous infusion maintains relatively stable concentrations, avoiding these transient peak insults. Notably, despite variations in nephrotoxicity definitions across studies, the protective effect remained highly consistent, reinforcing the robustness of the conclusion. Vancomycin-associated nephrotoxicity may present as acute interstitial nephritis or acute tubular injury, and continuous infusion may represent a strategy to reduce its occurrence.

No significant difference in mortality was observed between the two groups (RR = 1.05, 95% CI: 0.85–1.30, *P* = 0.629). This finding is not unexpected, as mortality in critically ill patients is influenced by multiple factors, including disease severity, underlying comorbidities, and source control; optimizing vancomycin administration alone may be insufficient to alter this hard endpoint. Nevertheless, this null result also suggests that the benefits of continuous infusion in terms of target attainment and nephrotoxicity reduction were not achieved at the expense of an increased risk of death.

The findings of this study have important implications for clinical practice. First, continuous vancomycin infusion should be considered a preferred strategy for achieving therapeutic targets in critically ill patients, particularly in those who require rapid attainment of therapeutic concentrations and minimization of nephrotoxicity risk. Second, continuous infusion reduces the frequency of blood sampling and dose adjustments, simplifies therapeutic drug monitoring, and may lower healthcare resource utilization. Third, the subgroup analyses in the pediatric population yielded results consistent with those in adults, supporting the use of continuous infusion strategies in children and neonates.

This study has several limitations. First, the majority of included studies were observational in design, which carries inherent risks of selection bias and confounding. Although subgroup analyses demonstrated that findings from RCTs were consistent with those from observational studies, residual confounding cannot be entirely excluded. Second, heterogeneity in vancomycin dosing regimens, target concentration ranges, and nephrotoxicity definitions across studies may limit the comparability of the pooled results. Third, only seven studies reported AUC/MIC outcomes, limiting the statistical power of this analysis. Fourth, publication bias was detected for serum concentration attainment (Egger *P* = 0.001); however, the effect estimate remained significant after trim-and-fill correction, supporting the robustness of the finding.

Several directions for future research merit consideration. First, large-scale, well-designed randomized controlled trials are needed to further validate the clinical benefits of continuous infusion. Second, the optimal target concentration ranges for different patient populations, including those with augmented renal clearance, obesity, or those undergoing renal replacement therapy RRT, remain to be determined. Third, the impact of shorter time to target attainment on patient-centered outcomes, such as time to bacteremia clearance and length of hospital stay, warrants further investigation. Fourth, Several included studies employed a historical control design, comparing later-era CIV patients with earlier-era IIV patients. This may introduce period-effect bias, as improvements in TDM practice and critical care over time could independently favor CIV outcomes. Although sensitivity analyses excluding serious risk of bias studies confirmed the robustness of our primary findings, this limitation should be considered when interpreting the results. Fifth, the pediatric studies combined neonates with older children, who differ in vancomycin PK and dosing requirements. However, all included pediatric studies used age- and weight- adjusted regimens based on validated PK models, which inherently controlled for developmental differences within each study. Subgroup analyses by age were not feasible due to the limited number of pediatric studies. This age-related heterogeneity should be considered when interpreting the pediatric results. Sixth, variability in loading dose practices across studies may represent a potential confounder, this should be considered when interpreting early target attainment outcomes. Seventh, the AUC/MIC ≥ 400 target used in this meta-analysis is primarily validated for MRSA infections and may not fully apply to other gram-positive pathogens. However, the included studies encompassed a range of pathogens. Furthermore, MIC distributions differ substantially across species, and the common assumption of MIC = 1 mg/L in studies without measured MICs may introduce species-dependent misestimation of exposure. This pathogen-level heterogeneity should be considered when interpreting the results. Due to insufficient pathogen-specific outcome data across studies, we were unable to perform a subgroup analysis restricted to S. aureus/MRSA infections. Finally, a cost-effectiveness analysis of continuous infusion is warranted from a health economics perspective.

## 5 Conclusions

This systematic review and meta-analysis demonstrates that, compared with intermittent infusion, continuous vancomycin infusion significantly improves serum concentration and AUC/MIC attainment, shortens the time to target attainment, and reduces the risk of nephrotoxicity, with no significant difference in mortality between the two groups. These findings remained generally robust across subgroup and sensitivity analyses. Future large-scale, well-designed RCTs are warranted to further validate these findings and to evaluate patient-centered clinical outcomes.

## Data Availability

The original contributions presented in the study are included in the article/Supplementary Material, further inquiries can be directed to the corresponding author/s.
