# Supplementary material for: Continuous vs. intermittent vancomycin infusion in critically ill patients: a systematic review and meta-analysis
Source: Front Med (Lausanne). 2026 Oct 13;13:1951699. doi: 10.3389/fmed.2026.1951699 (PMC13710284; doi:10.3389/fmed.2026.1951699)
Supplement: Supplementary file 1 [file Datasheet1.pdf]

Supplementary Material

1 Supplementary Figures and Tables

1.1 Supplementary Figures

Supplementary Figure 1. Risk of bias assessment of non-randomized studies using the ROBINS-I tool.

Supplementary Figure 2. Forest plot of serum concentration attainment by study design (RCT vs non- randomized studies).

Supplementary Figure 3. Forest plot of serum concentration attainment by patient population (children vs adults).

Supplementary Figure 4. Leave- one- out sensitivity analysis forest plots for binary outcomes. (A) Serum concentration attainment; (B) AUC/MIC attainment; (C) Nephrotoxicity; (D) Mortality;

Supplementary Figure 5. Leave- one- out sensitivity analysis forest plots for continuous outcomes. (A) Steady- state concentration; (B) AUC/MIC ratio; (C) Time

Supplementary Figure 6. Sensitivity analyses for binary outcomes excluding studies with serious risk of bias. (A)Serum concentration attainment; (B) AUC/MIC attainment; (C) Nephrotoxicity; (D) Mortality.

Supplementary Figure 7. Sensitivity analyses for continuous outcomes excluding studies with serious risk of bias. (A) Steady- state concentration; (B) AUC/MIC ratio; (C) Time to target attainment.

1.2 Supplementary Tables

Supplementary Table 1. PRISMA 2020 Checklist.

Supplementary Table 2. Details of the Search Strategy According to the Database.

Supplementary Table 3. Summary of findings and certainty of evidence for continuous versus intermittent vancomycin infusion using the GRADE approach.

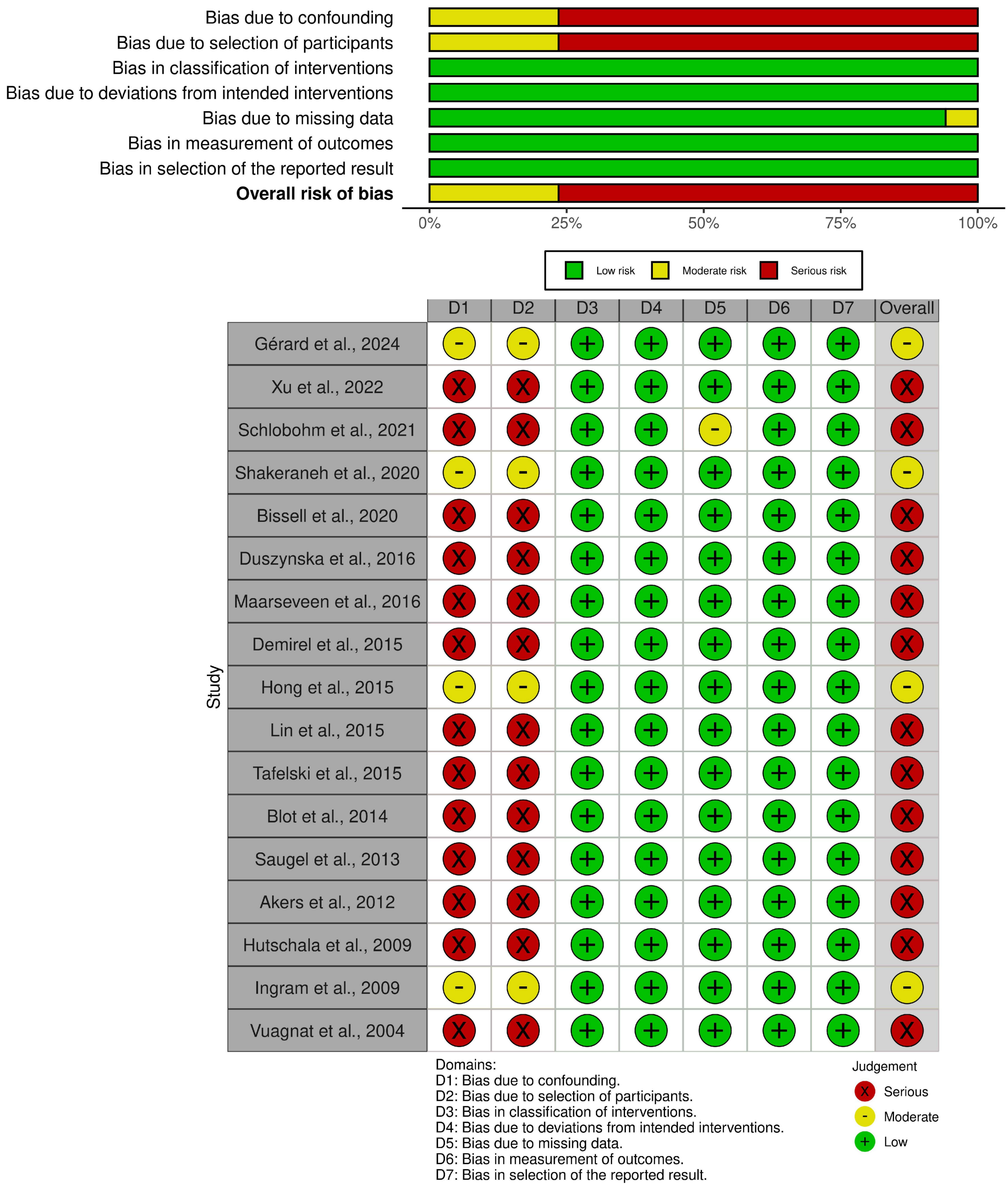

Supplementary Figure 1. Risk of bias assessment of non-randomized studies using the ROBINS-I tool.

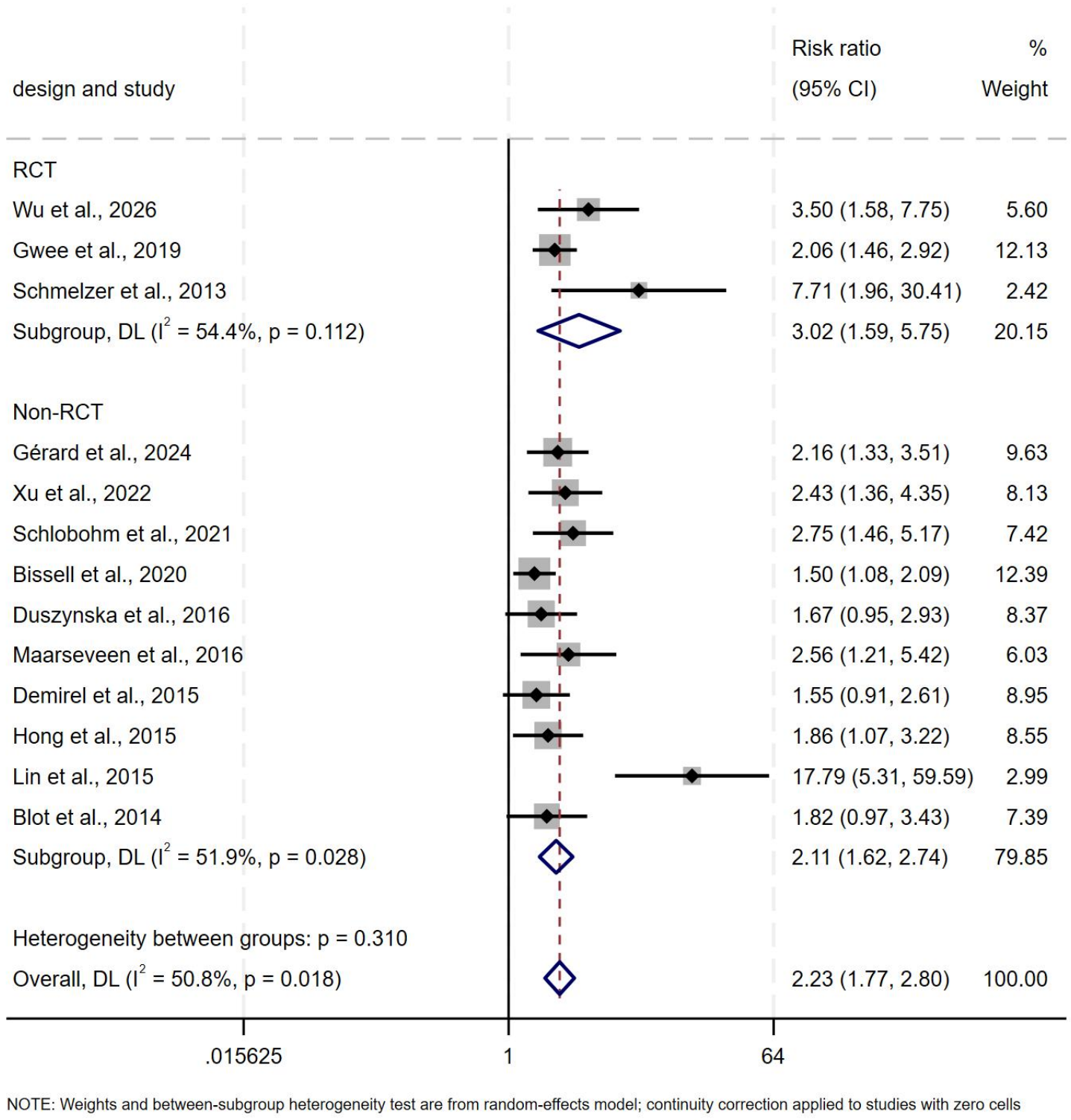

Supplementary Figure 2. Forest plot of serum concentration attainment by study design (RCT vs non- randomized studies).

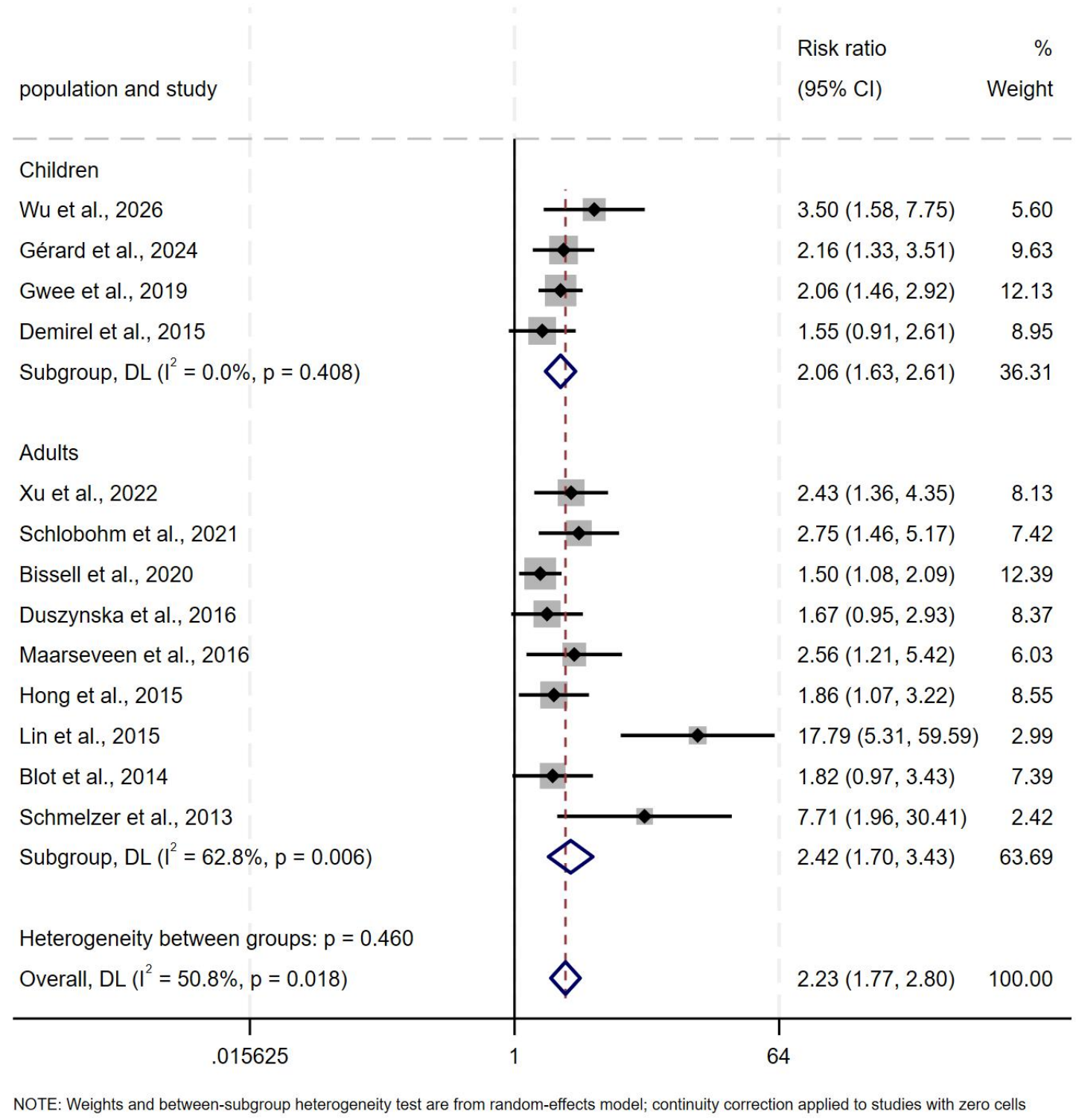

Supplementary Figure 3. Forest plot of serum concentration attainment by patient population (children vs adults).

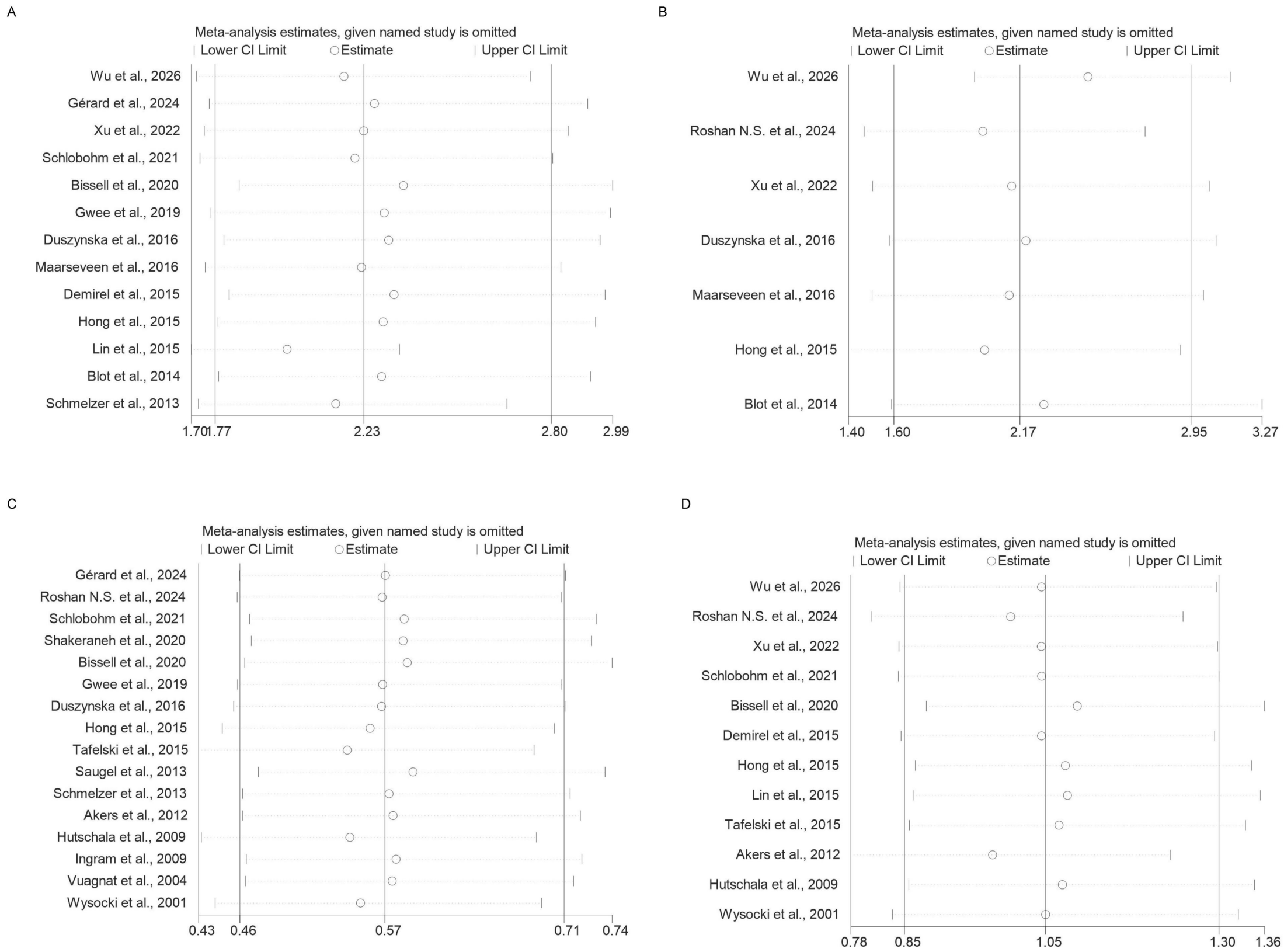

Supplementary Figure 4. Leave-one-out sensitivity analysis forest plots for binary outcomes. (A) Serum concentration attainment; (B) AUC/MIC attainment; (C) Nephrotoxicity; (D) Mortality.

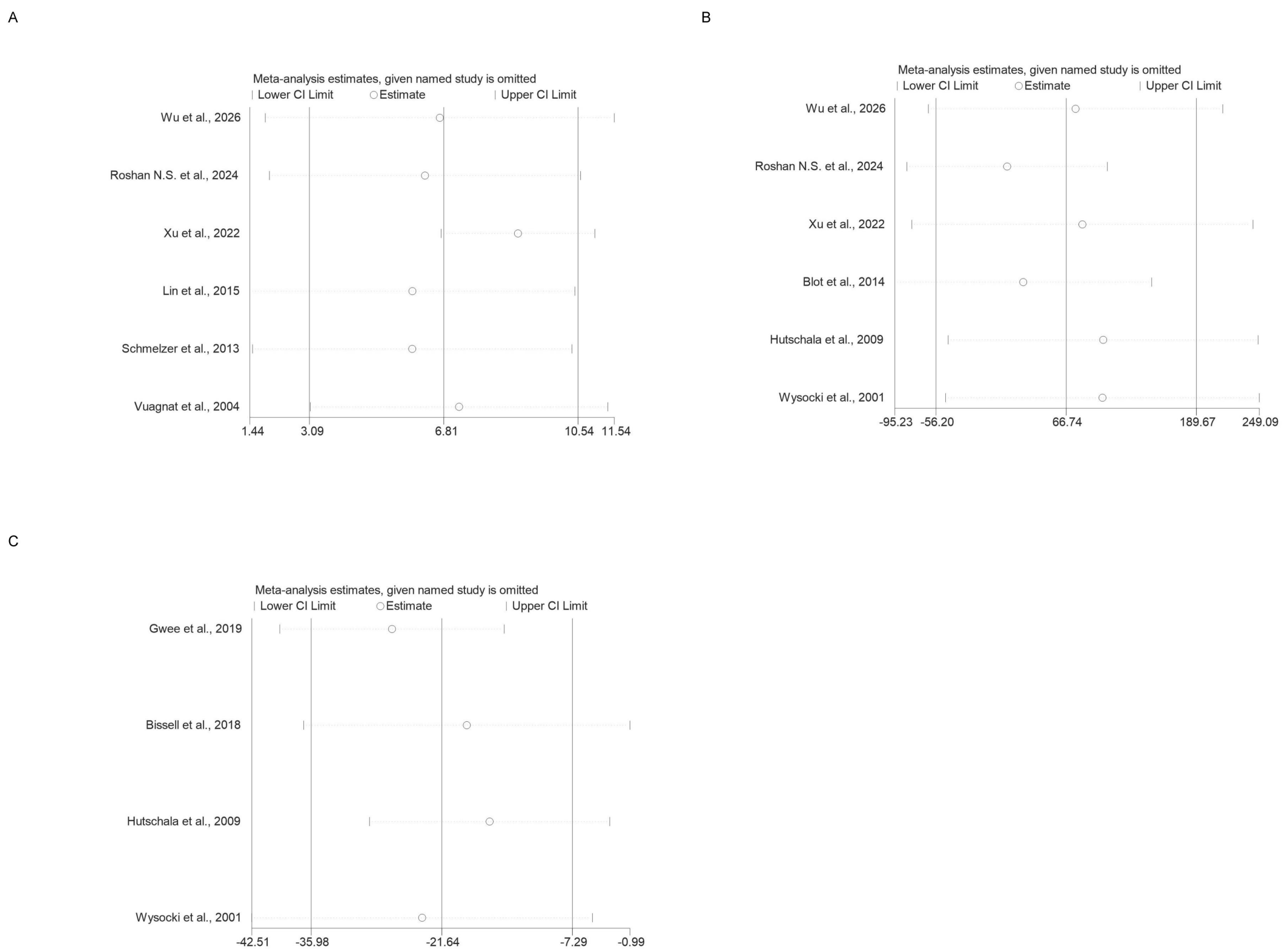

Supplementary Figure 5. Leave-one-out sensitivity analysis forest plots for continuous outcomes. (A) Steady-state concentration; (B) AUC/MIC ratio; (C) Time to target attainment.

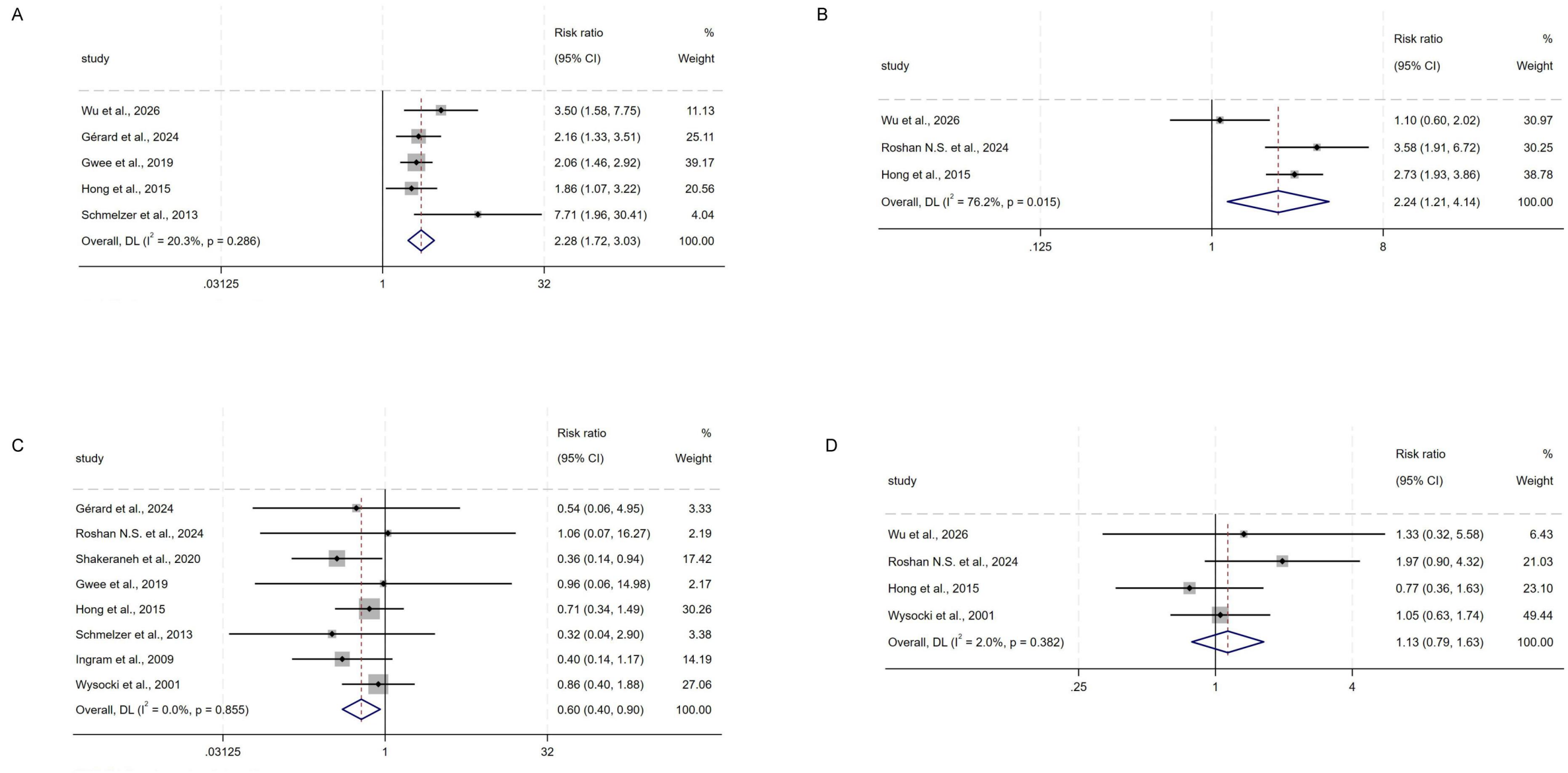

Supplementary Figure 6. Sensitivity analyses for binary outcomes excluding studies with serious risk of bias. (A)Serum concentration attainment; (B) AUC/MIC attainment; (C) Nephrotoxicity; (D) Mortality.

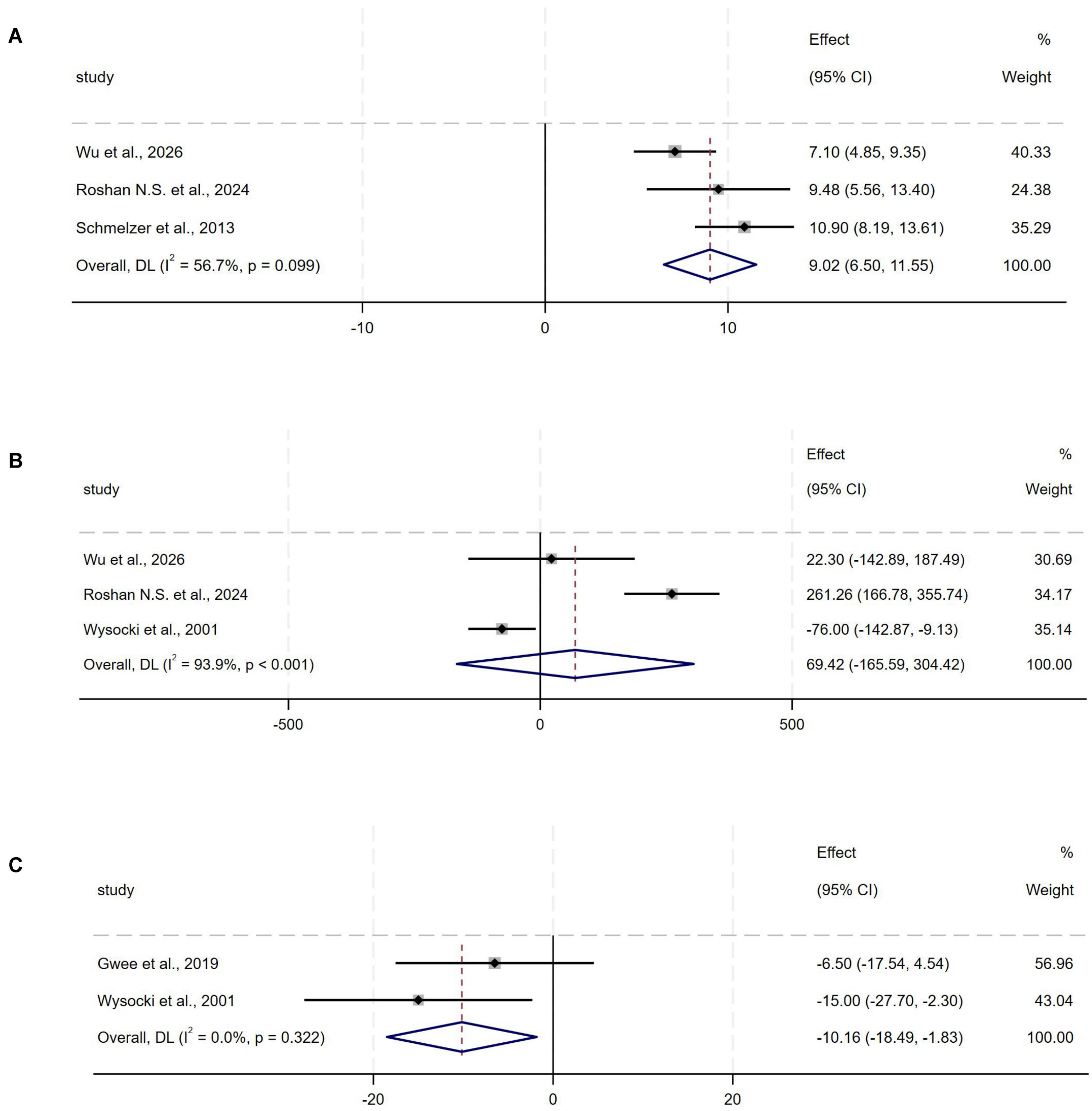

Supplementary Figure 7. Sensitivity analyses for continuous outcomes excluding studies with serious risk of bias. (A) Steady- state concentration; (B) AUC/MIC ratio; (C) Time to target attainment.

Supplementary Table 1. PRISMA 2020 Checklist.

Section and Topic	Item #	Checklist item	Location where item is reported
**TITLE**			
Title	1	Identify the report as a systematic review.	P1
**ABSTRACT**			
Abstract	2	See the PRISMA 2020 for Abstracts checklist.	P2
**INTRODUCTION**			
Rationale	3	Describe the rationale for the review in the context of existing knowledge.	P3
Objectives	4	Provide an explicit statement of the objective(s) or question(s) the review addresses.	P4
**METHODS**			
Eligibility criteria	5	Specify the inclusion and exclusion criteria for the review and how studies were grouped for the syntheses.	P5
Information sources	6	Specify all databases, registers, websites, organisations, reference lists and other sources searched or consulted to identify studies. Specify the date when each source was last searched or consulted.	P5
Search strategy	7	Present the full search strategies for all databases, registers and websites, including any filters and limits used.	P5
Selection process	8	Specify the methods used to decide whether a study met the inclusion criteria of the review, including how many reviewers screened each record and each report retrieved, whether they worked independently, and if applicable, details of automation tools used in the process.	P5
Data collection process	9	Specify the methods used to collect data from reports, including how many reviewers collected data from each report, whether they worked independently, any processes for obtaining or confirming data from study investigators, and if applicable, details of automation tools used in the process.	P6
Data items	10a	List and define all outcomes for which data were sought. Specify whether all results that were compatible with each outcome domain in each study were sought (e.g. for all measures, time points, analyses), and if not, the methods used to decide which results to collect.	P5
	10b	List and define all other variables for which data were sought (e.g. participant and intervention characteristics, funding sources). Describe any assumptions made about any missing or unclear information.	P6
Study risk of bias assessment	11	Specify the methods used to assess risk of bias in the included studies, including details of the tool(s) used, how many reviewers assessed each study and whether they worked independently, and if applicable, details of automation tools used in the process.	P6
Effect measures	12	Specify for each outcome the effect measure(s) (e.g. risk ratio, mean difference) used in the synthesis or presentation of results.	P7
Synthesis methods	13a	Describe the processes used to decide which studies were eligible for each synthesis (e.g. tabulating the study intervention characteristics and comparing against the planned groups for each synthesis (item #5)).	P7
	13b	Describe any methods required to prepare the data for presentation or synthesis, such as handling of missing summary statistics, or data conversions.	P7
	13c	Describe any methods used to tabulate or visually display results of individual studies and syntheses.	P7
	13d	Describe any methods used to synthesize results and provide a rationale for the choice(s). If meta-analysis was performed, describe the model(s), method(s) to identify the presence and extent of statistical heterogeneity, and software package(s) used.	P7
	13e	Describe any methods used to explore possible causes of heterogeneity among study results (e.g. subgroup analysis, meta-regression).	P7
	13f	Describe any sensitivity analyses conducted to assess robustness of the synthesized results.	P7
Reporting bias assessment	14	Describe any methods used to assess risk of bias due to missing results in a synthesis (arising from reporting biases).	P6
Certainty assessment	15	Describe any methods used to assess certainty (or confidence) in the body of evidence for an outcome.	P7

PRISMA 2020 Checklist

Section and Topic	Item #	Checklist item	Location where item is reported
**RESULTS**			
Study selection	16a	Describe the results of the search and selection process, from the number of records identified in the search to the number of studies included in the review, ideally using a flow diagram.	P8
	16b	Cite studies that might appear to meet the inclusion criteria, but which were excluded, and explain why they were excluded.	P8
Study characteristics	17	Cite each included study and present its characteristics.	P9
Risk of bias in studies	18	Present assessments of risk of bias for each included study.	P12
Results of individual studies	19	For all outcomes, present, for each study: (a) summary statistics for each group (where appropriate) and (b) an effect estimate and its precision (e.g. confidence/credible interval), ideally using structured tables or plots.	P9
Results of syntheses	20a	For each synthesis, briefly summarise the characteristics and risk of bias among contributing studies.	P12
	20b	Present results of all statistical syntheses conducted. If meta-analysis was done, present for each the summary estimate and its precision (e.g. confidence/credible interval) and measures of statistical heterogeneity. If comparing groups, describe the direction of the effect.	P13
	20c	Present results of all investigations of possible causes of heterogeneity among study results.	P13
	20d	Present results of all sensitivity analyses conducted to assess the robustness of the synthesized results.	P18
Reporting biases	21	Present assessments of risk of bias due to missing results (arising from reporting biases) for each synthesis assessed.	P18
Certainty of evidence	22	Present assessments of certainty (or confidence) in the body of evidence for each outcome assessed.	P19
**DISCUSSION**			
Discussion	23a	Provide a general interpretation of the results in the context of other evidence.	P19
	23b	Discuss any limitations of the evidence included in the review.	P21
	23c	Discuss any limitations of the review processes used.	P21
	23d	Discuss implications of the results for practice, policy, and future research.	P21
**OTHER INFORMATION**			
Registration and protocol	24a	Provide registration information for the review, including register name and registration number, or state that the review was not registered.	P5
	24b	Indicate where the review protocol can be accessed, or state that a protocol was not prepared.	P5
	24c	Describe and explain any amendments to information provided at registration or in the protocol.	NA
Support	25	Describe sources of financial or non-financial support for the review, and the role of the funders or sponsors in the review.	P23
Competing interests	26	Declare any competing interests of review authors.	P23
Availability of data, code and other materials	27	Report which of the following are publicly available and where they can be found: template data collection forms; data extracted from included studies; data used for all analyses; analytic code; any other materials used in the review.	P23

From: Page MJ, McKenzie JE, Bossuyt PM, Boutron I, Hoffmann TC, Mulrow CD, et al. The PRISMA 2020 statement: an updated guideline for reporting systematic reviews. BMJ 2021;372:n71. doi: 10.1136/bmj.n71

For more information, visit: http://www.prisma-statement.org/

Supplementary Table 2. Details of the Search Strategy According to the Database

Pubmed	("Vancomycin"[MeSH Terms] OR "Vancomycin"[Title/Abstract]) AND ("infusions, intravenous"[MeSH Terms] OR "Drug Administration Schedule"[MeSH Terms] OR "infusion*"[Title/Abstract] OR "intravenous"[Title/Abstract] OR "IV"[Title/Abstract] OR "administration"[Title/Abstract] OR "dosing"[Title/Abstract]) AND ("continuous"[Title/Abstract] OR "continuously"[Title/Abstract] OR "continuous infusion"[Title/Abstract] OR "intermitten*"[Title/Abstract] OR "bolus"[Title/Abstract] OR "intermittent infusion"[Title/Abstract] OR "pulse"[Title/Abstract]) AND ("randomized controlled trial"[Publication Type] OR "controlled clinical trial"[Publication Type] OR "randomized"[Title/Abstract] OR "randomised"[Title/Abstract] OR "placebo"[Title/Abstract] OR "clinical trial"[Title/Abstract] OR "clinical trials as topic"[MeSH Terms] OR "randomly"[Title/Abstract] OR "trial"[Title/Abstract] OR "Comparative Study"[Publication Type] OR "cohort"[Title/Abstract] OR "prospective"[Title/Abstract])
Embase	('vancomycin'/exp OR vancomycin:ti,ab) AND ('intravenous drug administration'/exp OR 'drug infusion'/exp OR infusion:ti,ab OR intravenous:ti,ab OR iv:ti,ab OR administration:ti,ab OR dosing:ti,ab) AND (continuous:ti,ab OR continuously:ti,ab OR 'continuous infusion':ti,ab OR intermitten*:ti,ab OR bolus:ti,ab OR 'intermittent infusion':ti,ab OR pulse:ti,ab) AND ('randomized controlled trial'/exp OR 'controlled study'/exp OR 'cohort analysis'/exp OR 'prospective study'/exp OR 'comparative study'/exp OR random*:ti,ab OR controlled:ti,ab OR cohort:ti,ab OR prospective:ti,ab OR comparative:ti,ab)
Cochrane	(vancomycin* OR "vancomycin"):ti,ab,kw AND (continuous OR continuously OR intermittent* OR bolus OR pulse):ti,ab,kw AND (infusion* OR intravenous OR iv OR administration OR dosing):ti,ab,kw
Web of Science	TS=(vancomycin) AND TS=(continuous OR "continuous infusion" OR intermitten* OR "intermittent infusion" OR bolus) AND TS=(infusion* OR intravenous OR IV OR administration OR dosing) AND TS=(random* OR "controlled trial" OR "clinical trial" OR cohort OR prospective OR retrospective)

Supplementary Table 3. Summary of findings and certainty of evidence for continuous versus intermittent vancomycin infusion using the GRADE approach.

Certainty assessment							№ of patients		Effect		Certainty	Importance
№ of studies	Study design	Risk of bias	Inconsistency	Indirectness	Imprecision	Other considerations	[[intervention]	[comparison]	Relative (95% CI)	Absolute (95% CI)		
**Serum concentration attainment**												
13	randomised trials	serious[a]	not serious	not serious	not serious	none	303/482 (62.9%)	143/515 (27.8%)	**RR 2.23** (1.77 to 2.80)	**342 more per 1,000** (from 214 more to 500 more)	⊕⊕⊕◯ Moderate[a]	CRITICAL
**AUC/MIC attainment**												
7	randomised trials	serious[a]	not serious	not serious	not serious	none	155/227 (68.3%)	65/219 (29.7%)	**RR 2.17** (1.60 to 2.95)	**347 more per 1,000** (from 178 more to 579 more)	⊕⊕⊕◯ Moderate[a]	IMPORTANT
**Nephrotoxicity**												
16	randomised trials	serious[a]	not serious	not serious	not serious	none	128/911 (14.1%)	168/752 (22.3%)	**RR 0.57** (0.46 to 0.71)	**96 fewer per 1,000** (from 121 fewer to 65 fewer)	⊕⊕⊕◯ Moderate[a]	CRITICAL
**Mortality**												
12	randomised trials	serious[a]	not serious	not serious	not serious	none	155/662 (23.4%)	130/577 (22.5%)	**RR 1.05** (0.85 to 1.30)	**11 more per 1,000** (from 34 fewer to 68 more)	⊕⊕⊕◯ Moderate[a]	IMPORTANT
**Steady-state concentration**												
6	randomised trials	serious[a]	serious[b]	not serious	not serious	none			**MD 6.81** (3.09 to 10.54)	**-- per 1,000** (from -- to --)	⊕⊕◯◯ Low[a,b]	IMPORTANT
**AUC/MIC ratio**												
6	randomised trials	serious[a]	serious[b]	not serious	not serious	none			**MD 66.74** (-56.20 to 189.67)	**-- per 1,000** (from -- to --)	⊕⊕◯◯ Low[a,b]	IMPORTANT
**Time to target attainment**												
4	randomised trials	serious[a]	serious[b]	not serious	not serious	none			**MD -21.64** (-35.98 to -7.29)	**-- per 1,000** (from -- to --)	⊕⊕◯◯ Low[a,b]	NOT IMPORTANT

CI: confidence interval; **RR:** risk ratio

Explanations

a. The majority of included studies were non-randomized retrospective cohort designs with inherent selection bias and confounding
b. Substantial statistical heterogeneity ($I^2 > 50\%$)
